# Circulating MicroRNAs in Testicular Germ Cell Tumors: Biomarkers for Diagnosis, Surveillance, and Treatment Stratification

**DOI:** 10.3390/jpm16080400

**Published:** 2026-07-27

**Authors:** Stamatios Katsimperis, Lazaros Tzelves, Patrick Juliebø-Jones, Andreas Skolarikos

**Affiliations:** 1 Second Department of Urology, National and Kapodistrian University of Athens, Sismanogleio Hospital, 15126 Athens, Greece; 2 Department of Urology, Haukeland University Hospital, 5009 Bergen, Norway

**Keywords:** testicular germ cell tumors, miR-371a-3p, circulating microRNAs, liquid biopsy, serum tumor markers

## Abstract

Testicular germ cell tumors (TGCTs) are highly curable malignancies, yet their clinical management still relies heavily on conventional serum tumor markers, including alpha-fetoprotein, beta-human chorionic gonadotropin, and lactate dehydrogenase, which have limited sensitivity and specificity in several clinically relevant settings. Circulating microRNAs, particularly miR-371a-3p, have emerged as promising liquid biopsy biomarkers with potential applications across the TGCT disease continuum. This narrative review summarizes the current evidence on the biological basis, diagnostic performance, clinical utility, and limitations of circulating microRNAs in TGCTs. MiR-371a-3p demonstrates high diagnostic accuracy for viable non-teratomatous TGCTs and consistently outperforms classical serum tumor markers in primary diagnosis. Its rapid decline after effective treatment and its association with tumor burden support potential roles in chemotherapy monitoring, early relapse detection during surveillance, and the assessment of selected post-chemotherapy residual masses. However, its inability to detect teratoma remains a major biological limitation, particularly in non-seminomatous residual disease and surveillance settings. Additional barriers to implementation include assay heterogeneity, the lack of universally accepted cutoffs, the variable use of serum versus plasma, and the absence of broad regulatory approval. Emerging translational data suggest that miR-371a-3p may also contribute to tumor–microenvironment communication and cisplatin resistance, although these findings remain preclinical. Overall, miR-371a-3p represents one of the most promising biomarkers in TGCT management, but its routine clinical integration will require standardized analytical protocols and prospective validation in marker-guided decision pathways.

## 1. Introduction

Testicular germ cell tumors are the predominant malignancy in males between 15 and 40 years of age, with incidence rates of 7–10 per 100,000 in Western populations [1]. Although cure rates now exceed 90% owing to cisplatin-based combination chemotherapy and risk-adapted surgery, the management of individual patients continues to depend heavily on three protein-based serum tumor markers: AFP, β-hCG, and LDH [2]. These markers carry well-documented limitations. In pure seminoma, AFP is definitionally absent, while β-hCG and LDH are elevated in only approximately 28% and 29% of patients, respectively. In non-seminomatous germ cell tumors (NSGCTs), AFP, β-hCG, and LDH are elevated in roughly 60%, 53%, and 39% of cases. Consequently, a large proportion of patients receive treatment decisions based on imaging and histology alone, without a sensitive biochemical correlate of tumor activity.

Precision oncology requires markers that are tumor-specific, biologically rational, sensitive at low disease burden, and responsive to treatment in real time. The classical TGCT markers satisfy some of these criteria but fall short on specificity. LDH reflects cell death broadly, AFP rises in hepatocellular dysfunction, and β-hCG lacks specificity for germ cell malignancy, as it may be elevated through several mechanisms unrelated to viable testicular tumors. These include pituitary-derived hCG secretion following the loss of gonadal steroid feedback in hypogonadal states, a scenario directly relevant to orchiectomized testicular cancer patients; ectopic production by non-trophoblastic malignancies, notably bladder, lung, gastrointestinal, pancreatic, and neuroendocrine tumors; and, less commonly, marijuana use and analytical interference from heterophile antibodies [3,4,5]. Numerous candidate markers have been investigated over the years without achieving clinical implementation [6].

Members of the miR-371~373 microRNA cluster have emerged as credible alternatives over the past decade. Germ cell tumors recapitulate features of pluripotent embryonic stem cells (ESCs), including the expression of ESC-specific miRNA clusters, of which miR-371~373 is characteristic [7]. The most diagnostically relevant member, miR-371a-3p, is detectable in the peripheral circulation of TGCT patients, correlates with tumor burden, declines rapidly following effective treatment, and is elevated in both seminoma and NSGCT, addressing a key gap left by the classical markers [8]. This review evaluates the evidence for miR-371a-3p as a precision liquid biopsy analyte across the clinical spectrum of TGCT management and examines recent discoveries about its biological function that may hold therapeutic relevance.

## 2. Methods

For this narrative review, the literature was searched in PubMed/MEDLINE, Scopus, and Google Scholar for relevant English-language articles published through March 2026. The literature search and study screening were performed independently by three authors (S.K., L.T., P.J.); disagreements over inclusion were resolved by discussion, with the senior author (A.S.) arbitrating where consensus was not reached. The search strategy used combinations of the following terms: “testicular germ cell tumor”, “TGCT”, “microRNA”, “miR-371a-3p”, “miR-371”, “liquid biopsy”, “biomarker”, “surveillance”, “residual disease”, “chemotherapy monitoring”, “cisplatin resistance”, and “extracellular vesicles.” Additional studies were identified by screening the reference lists of selected articles. Priority was given to original clinical studies, prospective cohorts, translational research, and recent review articles with direct relevance to the role of circulating microRNAs in the diagnosis, surveillance, treatment monitoring, and biological characterization of TGCTs. When multiple studies from the same group or dataset overlapped, priority was given to the most recent or methodologically rigorous report. Studies were included if they reported original clinical, prospective, translational, or recent review data on circulating microRNAs in TGCT diagnosis, surveillance, treatment monitoring, or biology. Conference abstracts were included only where no peer-reviewed equivalent existed; non-English reports and studies without extractable outcome data were excluded. Given the narrative design of the review, no formal systematic study selection process or risk-of-bias assessment was performed; readers should interpret the synthesis accordingly.

## 3. Biological Basis: Why miR-371a-3p in Germ Cell Tumors?

### 3.1. The Embryonic Stem Cell Connection

MicroRNAs are small (~22 nucleotide) non-coding RNAs that regulate gene expression post-transcriptionally. The miR-371~373 cluster is a hallmark of undifferentiated human ESCs and is downregulated during lineage commitment [7]. Because TGCTs arise from primordial germ cells and retain an embryonic gene expression program, they overexpress this cluster, and this overexpression is measurable in serum. Of the cluster members, miR-371a-3p consistently outperforms miR-372-3p, miR-373-3p, and miR-367-3p, achieving superior receiver operating characteristic performance in every comparative study [9]. It should be emphasized that miR-371a-3p functions clinically as a circulating (serum or plasma) biomarker; although it is demonstrably expressed at the tissue level, its utility rests on non-invasive blood measurement rather than on assay of the pathological specimen itself. Unlike broad liquid-biopsy approaches applied across many solid tumors, circulating miR-371a-3p is comparatively tumor-restricted—its diagnostic value derives from the embryonic stem-cell program specific to germ cell tumors—with the principal non-malignant confounder being pregnancy. Two notable biological limitations apply to all cluster members. First, the miR-371~373 cluster is not expressed in teratoma, the differentiated histological subtype, which does not maintain the pluripotent ESC program. Second, although miR-371a-3p is highly expressed in germ cell neoplasia in situ (GCNIS) cells at the tissue level, it cannot reliably be detected in serum from men with GCNIS in the absence of an invasive tumor: approximately half of men with GCNIS showed elevated serum levels, and detection appears to require a threshold number of GCNIS cells [10]. Consequently, circulating miR-371a-3p is not suitable for screening patients at risk of the precursor lesion, and testicular biopsy with immunohistological examination remains the most accurate method for diagnosing GCNIS [10].

### 3.2. Extracellular Vesicle-Mediated Release and Tumor Microenvironment Effects

Recent cell culture and clinical tissue studies have begun to clarify the mechanism of miR-371a-3p secretion and its functional consequences within the tumor ecosystem. TGCT cells actively release extracellular vesicles (EVs) enriched with oncogenic miRNAs; among 165 differentially expressed miRNAs identified in TGCT-derived versus normal testicular fibroblast EVs, miR-371a-3p and miR-371a-5p were the most consistently upregulated [11]. These EVs are internalized by stromal cells of the tumor microenvironment (TME), transferring oncogenic miRNA cargo. Co-cultivation of fibroblast lines with five distinct TGCT cell lines produced a mean 982-fold increase in intracellular miR-371a-3p in the recipient fibroblasts [11]. Functional consequences in recipient cells included enhanced collagen contraction in fibroblasts and increased angiogenic activity in endothelial cells, phenotypes consistent with tumor-supportive stromal remodeling, although the in vivo relevance of these observations remains to be established.

Clinical translation of these findings confirmed that EVs released by non-teratomatous TGCT tissue explants contain significantly elevated miR-371~373 cluster members compared to adjacent normal parenchyma and that miRNA levels in EVs correlate inversely with the degree of tumor differentiation [12]. When TGCT cells were experimentally differentiated with all-trans retinoic acid, the miR-371~373 cluster declined and let-7e, associated with differentiated states, increased, both intracellularly and in secreted EVs. This reciprocal relationship suggests that the EV-derived miRNA profile could serve as a differentiation index, although, from a purely diagnostic standpoint, measuring total circulating miR-371a-3p in serum remains superior to quantifying EV-bound fractions [12].

### 3.3. A Murine Model Supporting Translational Research

The murine miR-290~295 cluster is orthologous to the human miR-371~373 cluster. In the gPAK transgenic mouse model, in which tumors histologically comparable to human mixed non-seminomas develop spontaneously, the murine orthologs miR-291a-3p, miR-292-3p, and miR-293 are strongly expressed in embryonal carcinoma (EC) cell lines and detectable in serum exclusively in mice with EC-containing tumors [13]. Experimentally induced differentiation suppressed cluster expression and elevated cell cycle inhibitors Cdkn1a, Rbl2, and Lats2, indicating that the cluster promotes proliferation by suppressing G1/S checkpoint regulators in the undifferentiated state. While the gPAK model provides a potentially useful platform for preclinical drug testing and early detection strategy development, differences between murine and human tumor biology warrant caution when extrapolating results directly to clinical settings [13].

## 4. Diagnostic Performance and Individualized Evaluation of Testicular Masses

### 4.1. Primary Diagnosis

Twelve studies encompassing over 1600 TGCT patients have evaluated miR-371a-3p for primary diagnosis, consistently reporting area under the receiver operating characteristic curve (AUC) values of 0.93–0.98, sensitivity of 84.7–96%, and specificity of 84.7–100% [8,9,14]. This performance exceeds that of any individual classical serum tumor marker (STM) and is not improved by combining miR-371a-3p with other cluster members in panels. The most important prospective dataset comes from a multicenter study of 616 TGCT patients and 258 controls, which reported an AUC of 0.953 for non-metastatic disease and 0.996 for metastatic cases [14]. Table 1 summarizes key study-level data.

An important caveat applies to the interpretation of these pooled figures: the studies summarized in Table 1 differ in several methodological respects that limit direct cross-study comparisons. These include the use of serum versus plasma, the choice of qRT-PCR versus ddPCR platforms, variation in endogenous normalization controls and positivity thresholds, and differences in cohort composition (disease stage, histological spectrum, and proportion of low-volume tumors). Case–control study designs, which predominate in this literature, are also susceptible to spectrum bias and may overestimate diagnostic accuracy relative to consecutive clinical cohorts. The figures cited should therefore be interpreted as indicative of strong performance rather than as precisely poolable estimates.

Two analytical approaches have been validated: quantitative reverse transcriptase PCR (qRT-PCR) with preamplification, currently the standard, and digital droplet PCR (ddPCR), which enables absolute quantification without preamplification. A head-to-head comparison found concordant performance between the two methods, with ddPCR yielding a specificity of 100% and a sensitivity of 89% in 180 samples [15]. DdPCR offers logistical advantages in throughput and eliminates the preamplification variability that complicates qRT-PCR in low-abundance samples. Importantly, a recent real-world prospective study confirmed that these performance characteristics are achievable under routine clinical conditions, including on alternative thermocycler platforms, with a sensitivity of 90.9% and an AUC of 0.92 [16]. This study also demonstrated a strong positive correlation between preoperative miR-371a-3p levels and tumor diameter in non-metastatic patients (Spearman ρ = 0.74), and significantly higher marker levels in metastatic compared to non-metastatic disease, reinforcing the association between miR-371a-3p expression and tumor burden [16]. Accordingly, the pooled figures cited throughout should be read as indicative of a consistent direction of effect rather than as precise, poolable estimates, given the predominance of small and case–control cohorts.

**Table 1:** Performance characteristics of miR-371a-3p for primary TGCT diagnosis.

Author, Year	*N*	Sensitivity (%)	Specificity (%)	AUC	Matrix	Platform	Design
Dieckmann, 2019 [14]	616	91.8	96.1	0.97	Serum	qRT-PCR	Prospective multicenter
Nappi, 2019 [17]	110	96.0	100	0.97	Plasma	qRT-PCR	Prospective
van Agthoven, 2017 [18]	250	89.0	90.0	0.95	Serum	qRT-PCR	Retrospective
Badia, 2021 [19]	69	93.1	100	0.98	Serum	qRT-PCR	Retrospective
Myklebust, 2021 [15]	180	89.0	100	—	Serum	ddPCR vs. qRT-PCR	Prospective
Sequeira, 2022 [20]	82	93.6	100	0.98	Serum	ddPCR	Retrospective
Palermo, 2025 [16]	72	90.9	89.3	0.92	Serum	qRT-PCR	Prospective (real-world)

AUC = area under the receiver operating characteristic curve; ddPCR = digital droplet PCR; qRT-PCR = quantitative reverse transcriptase PCR.

### 4.2. Individualized Evaluation of Small and STM-Negative Testicular Masses

Refinements in scrotal ultrasound have increased the incidental detection of small testicular lesions, many of which prove to be benign on histology, including Leydig cell tumors, fibrotic nodules, and sarcoidosis. In patients with STM-negative lesions, current guidelines recommend testis-sparing surgery with intraoperative frozen section examination, a procedure that carries non-trivial morbidity and exposes patients with benign disease to an unnecessary orchiectomy risk. A tool that could non-invasively distinguish malignant from benign masses prior to surgery would meaningfully personalize this decision pathway.

A retrospective analysis of 641 patients, including 87 with sub-centimeter masses, found miR-371a-3p elevation in 44.1% of patients with small masses, compared to AFP, β-hCG, and LDH elevations of 2.3%, 11.6%, and 7.0%, respectively [21]. Sensitivity in NSGCTs remained near 100% regardless of lesion size. However, sensitivity in seminoma declines notably with decreasing tumor dimensions, falling to approximately 59% in lesions under 10 mm and 77% in those between 10 and 20 mm [14]. This size-dependent limitation in seminoma is clinically important: a negative miR-371a-3p result cannot safely exclude small seminomas, and histological confirmation via surgical exposure remains mandatory for all sonographically suspicious masses. Conversely, a positive result in a patient with an STM-negative mass provides supporting evidence for TGCT and may strengthen the clinical rationale for orchiectomy, although this management implication requires prospective validation before it can be broadly recommended.

## 5. Chemotherapy Monitoring and Personalized Response Assessment

Circulating miR-371a-3p has a serum half-life of under 24 h, substantially shorter than those of AFP (approximately five days) and β-hCG (approximately 24–36 h). This rapid clearance enables near-real-time assessment of the tumor-secreting cell mass during chemotherapy, a property that neither classical marker can match. Measuring STMs prior to each treatment cycle is already guideline-recommended; adding miR-371a-3p to this schedule could facilitate more granular, biologically informed monitoring [22].

A prospective multicenter study measured serial miR-371a-3p in 70 clinical stage IIA/IIB and 46 stage III patients undergoing standard cisplatin-based chemotherapy [14]. In stage IIA/IIB patients, marker levels declined significantly after the first cycle and plateaued thereafter; stage III patients showed further reductions after a second cycle, with the timing of nadir reflecting disease burden. Two patients whose levels increased rather than decreased during chemotherapy subsequently experienced fatal disease progression, an observation that, while based on a very small number of cases, suggests that a rising marker trajectory during treatment may identify non-responders who warrant close monitoring and potential regimen reassessment [14]. Complementary data confirm that marker decline within the first week of chemotherapy correlates with eventual complete response and that pre-chemotherapy miR-371a-3p elevation is associated with inferior progression-free and overall survival [23,24].

A caveat deserves explicit acknowledgment: not all patients have detectable miR-371a-3p after orchiectomy and before chemotherapy; one study found measurable levels in only half of 180 patients at this interval [24]. This likely reflects the lower residual tumor burden post-surgery and represents a genuine assay sensitivity limitation at low miRNA concentrations. Improving detection sensitivity in this low-burden setting is an active research priority before chemotherapy monitoring can be reliably implemented in routine practice.

## 6. Postchemotherapy Residual Masses: Personalizing the Surgical Decision

### 6.1. Non-Seminoma: Viable Tumor vs. Necrosis

Among metastatic NSGCT patients who achieve normalization of conventional STMs after first-line chemotherapy, approximately two-thirds harbor residual radiographic masses. Histopathological analysis of these lesions reveals viable cancer, teratoma, and necrosis in roughly 10%, 40%, and 50% of cases, respectively [22]. Postchemotherapy retroperitoneal lymph node dissection (pcRPLND) is currently recommended for residual masses exceeding one centimeter, yet roughly half of resected specimens contain only necrosis, meaning that these patients derive no oncological benefit from a procedure associated with significant surgical morbidity. Identifying which patients require surgery is therefore a high-priority personalized medicine question.

MiR-371a-3p shows preliminary promise in distinguishing viable residual tumor from necrosis, although recent prospective data have tempered initial expectations. An early retrospective study of 82 NSGCT patients prior to pcRPLND reported an AUC of 0.874 for viable tumor detection, with sensitivity and negative predictive value each reaching 100% for retroperitoneal masses up to three centimeters [25]. However, the largest prospective multicenter study to date, enrolling 180 patients undergoing postchemotherapy resection, reported a lower sensitivity of 68.9%, specificity of 99.3%, an AUC of 0.813, and an NPV of 0.905 [26]. Critically, this study demonstrated that sensitivity is significantly dependent on the proportion of viable cancer within the mass: only 33.3% of patients with ≤10% viable cancer had elevated marker levels, compared to 85.7% of those with >50% viable cancer [26]. Somatic-type malignancy arising from teratoma (n = 6) showed no marker expression. The authors propose that reduced vascularization in chemotherapy-regressed tissue limits miRNA drainage into the circulation, explaining why small islets of residual viable cancer in postchemotherapy masses may escape detection even when comparably sized primary tumors are identified accurately [26]. A separate cohort found that all 44 patients whose specimens contained only necrosis, fibrosis, or teratoma tested marker-negative prior to surgery, while three of four patients with viable cancer tested positive [23]. These results indicate that while a positive miR-371a-3p result carries a very high predictive value for viable cancer (PPV 0.969), a negative result does not reliably exclude it, particularly when the viable cancer component is small.

The critical limitation is the teratoma gap: miR-371a-3p is not expressed in teratoma, meaning that a negative result cannot exclude this histology. Since teratoma requires resection, being chemotherapy- and radiation-resistant, the teratoma gap prevents the marker from safely replacing pcRPLND in patients who might harbor teratomatous components. This limitation is not merely technical; it is fundamental to the biology of the marker and materially constrains its clinical utility in the postchemotherapy NSGCT setting. A related clinical scenario is growing teratoma syndrome, in which residual masses enlarge despite declining or normal classical serum tumor markers, reflecting the chemotherapy-driven differentiation of embryonal carcinoma into teratoma [10]. Neither classical markers nor miR-371a-3p are informative in this setting, underscoring the need for novel teratoma-specific biomarkers. A negative miR-371a-3p result may help identify the absence of viable malignant disease, but it cannot serve as a standalone decision tool when teratoma remains a realistic diagnostic possibility. Candidate teratoma markers, including serum miR-375 and comprehensive small RNA sequencing, have not yielded a clinically validated alternative [27,28]. A combination of hypermethylated RASSF1A cell-free DNA with miR-371a-3p has been proposed as a complementary panel with a sensitivity of 100% for TGCT, including teratoma, but requires independent validation [29]. Tissue-level markers AGR2 and KRT19, identified by transcriptomic analysis of pcRPLND specimens, showed 100% sensitivity and specificity for distinguishing teratoma from necrosis in an independent cohort and may eventually enable functional imaging-based detection [30].

### 6.2. Seminoma Residual Masses: An Alternative to FDG-PET?

In seminoma, postchemotherapy residual masses smaller than three centimeters are managed conservatively, as they predominantly contain necrotic tissue. For masses above three centimeters, current guidelines recommend FDG-PET, which carries a positive predictive value of only approximately 23%, generating frequent false-positive results and unnecessary additional treatment [31]. A pilot study of 23 patients with residual seminoma masses found that normal miR-371a-3p levels after chemotherapy reliably identified the absence of viable tumor, while elevated levels predicted living seminoma [32]. These findings are based on a small patient cohort and should be considered preliminary. Prospective validation in larger series is required before any role for miR-371a-3p as a complement or alternative to FDG-PET can be established in this setting.

## 7. Surveillance, Early Relapse Detection, and Precision Follow-Up

Active surveillance is the preferred management strategy for clinical stage I TGCT after orchiectomy in patients willing to adhere to a structured follow-up schedule. The objective of surveillance is the early detection of the roughly 15–20% of stage I patients who will relapse, while sparing the majority from unnecessary adjuvant treatment. Current schedules rely on periodic clinical examination, conventional STM measurement, and cross-sectional CT imaging. The cumulative radiation dose from repeated CT scans is clinically meaningful, particularly in a young population already at elevated risk of secondary malignancy, and the economic burden of high-frequency imaging is substantial [33]. MiR-371a-3p has the potential to reduce dependence on imaging by providing a biochemical relapse signal that precedes radiographic disease.

The evidence that miR-371a-3p is elevated at the time of macroscopic relapse is well established. In a prospective multicenter study, 38 of 46 relapsing patients showed elevated levels, yielding a sensitivity of 82.6%, a specificity of 96.1%, and an AUC of 0.921 for relapse detection; marker levels declined significantly following successful salvage treatment in the majority [14]. A retrospective analysis of 151 stage I patients found miR-371a-3p elevated in 94.1% at the time of relapse, compared to normal AFP and β-hCG in 62% of the same patients, illustrating the marker’s advantage over classical STMs at this clinical moment [34].

The most encouraging data for surveillance application come from a prospective series of 33 stage I patients followed with serial miR-371a-3p measurements during active surveillance [35]. Of the ten who relapsed, all showed marker elevation, and elevated levels were detected a median of two months (range, 0–5 months) earlier than by standard investigations, including imaging and classical STMs. No relapse was missed, and non-relapsing patients maintained normal values throughout, with a single transient exception. These figures, however, derive from a single small cohort with only ten relapse events; a larger plasma-based surveillance series reported markedly lower sensitivity for relapse detection (62.8%; Table 2), underscoring that the favorable lead-time signal has not yet been reproduced at scale or across matrices. However, it is important to distinguish between detecting relapse at the time it occurs and reliably predicting recurrence before conventional methods, related but distinct clinical questions. The former is well supported; the latter, while suggested by the lead-time data, requires confirmation in adequately powered prospective studies before surveillance protocols can be safely modified on this basis.

Uncertainty remains about whether elevated miR-371a-3p levels in the immediate postoperative period, before any clinical evidence of relapse, predict subsequent disease progression. A cohort of 151 patients found no association between postoperative marker elevation and future relapse, while another group reported significantly shorter relapse-free survival in patients with persistently elevated postoperative levels [34,36]. These contradictory findings likely reflect differences in the timing of blood collection, assay protocols, positivity thresholds, and cohort composition across studies. Until this discordance is resolved by prospective standardized trials with harmonized protocols, the predictive value of immediate postoperative miR-371a-3p levels remains uncertain, and conclusions about surveillance de-escalation based on marker levels alone should be considered premature. Table 2 summarizes the available relapse detection data.

For pure seminoma specifically, marker-guided surveillance represents an attractive personalized approach: were miR-371a-3p monitoring validated as the primary trigger for imaging, pure seminoma patients, whose only reliable classical marker is β-hCG, elevated in fewer than 30%, could potentially follow a substantially lighter imaging schedule. For NSGCT, the teratoma gap necessitates ongoing imaging to capture teratoma-only recurrences that escape marker detection. At present, miR-371a-3p should be considered an adjunctive relapse-detection biomarker rather than a replacement for imaging, particularly in NSGCT, where teratoma-only relapse remains marker-negative.

## 8. miR-371a-3p and Cisplatin Resistance: Preclinical Evidence for a Potential Therapeutic Target

The observation that miR-371a-3p is not merely a biomarker but an active contributor to TGCT biology has opened a distinct line of preclinical investigation with potential therapeutic implications. Cisplatin-based chemotherapy achieves high cure rates in TGCT, but a clinically important subset of patients develops or presents with cisplatin-refractory disease, for which salvage options are limited.

Cisplatin-resistant subclones derived from three parental NSGCT cell lines expressed miR-371a-3p at 2- to 230-fold higher levels than their cisplatin-sensitive parent lines [37]. Transfection of these resistant cells with antagomirs, synthetic miRNA antagonists, significantly restored cisplatin sensitivity, as evidenced by reduced cell viability and increased apoptosis rates following drug exposure. Notably, antagomir treatment also enhanced cisplatin sensitivity in previously sensitive cell lines, suggesting that miR-371a-3p suppression could potentially reduce the effective cisplatin dose required, a hypothesis worth exploring given the substantial long-term toxicity of cisplatin-based regimens [37].

These findings establish proof of concept for antagomir-based therapeutic targeting of miR-371a-3p in cisplatin-refractory TGCT. However, the entire evidence base is currently derived from in vitro models, and the translational pathway remains long. In vivo pharmacokinetic feasibility, miRNA-specific delivery systems, acceptable off-target profiles, and demonstration of efficacy in animal models are all required before clinical evaluation can be considered. The gPAK mouse model described in Section 3.3 provides one available platform for such preclinical investigation [13].

## 9. Beyond Oncology: Reproductive and Gestational Biology

The biological reach of miR-371a-3p extends beyond oncology in ways that are relevant both to the clinical interpretation of the diagnostic test and to understanding broader aspects of reproductive biology. The miR-371~373 cluster is expressed in placental tissue and primary trophoblasts, and a case–control study measuring miR-371a-3p in 36 third-trimester pregnant women detected the marker in all participants; it was undetectable in all 12 non-pregnant female controls [38]. Using the TGCT diagnostic cutoff, 30 of 36 pregnant women exceeded the positive threshold, though levels were significantly lower than those observed in patients with active tumor.

This finding has two direct implications. First, pregnancy represents a biological confounder that is important for assay interpretation and biomarker specificity studies: miR-371a-3p is physiologically elevated in pregnancy and can cross the standard TGCT positivity threshold, a consideration that would be relevant if the test were applied in clinical scenarios beyond male TGCT. Second, these findings extend the biology of the miR-371~373 cluster into reproductive medicine: the cluster is likely involved in placenta-mediated feto–maternal communication, consistent with existing knowledge that placental miRNAs packaged in EVs can influence distant organs via the maternal circulation [38].

Complementary evidence from male reproductive biology demonstrates that miR-371a-3p is detectable at high concentrations in seminal plasma and correlates positively with sperm concentration. Tissue-level analysis has localized its expression in healthy males exclusively to testicular and epididymal tissue, suggesting a functional role in spermatogenesis [39]. The precise mechanism remains to be defined, but the cumulative evidence positions miR-371a-3p as a molecule relevant to gonadal biology broadly, not only in the context of malignant transformation.

## 10. Technical Considerations and the Path to Clinical Standardization

The most significant barrier to routine clinical implementation of miR-371a-3p testing is the absence of a universally standardized, regulatory-approved assay. One commercially available CE-certified test exists (mir|detect, Bremerhaven, Germany), enabling quantification of miR-371a-3p expression in serum using qRT-PCR across standard real-time PCR platforms [8]. No equivalent test holds CLIA approval in the United States, restricting access outside research settings in that jurisdiction.

Several pre-analytical variables influence measured values and complicate cross-study comparisons: the choice of serum versus plasma (the normalizer miR-30b-5p behaves differently between matrices, and clotting factor interactions in plasma may reduce miR-371a-3p detection); hemolysis, which measurably alters miRNA levels; time from blood collection to processing; and freeze–thaw cycles during storage and shipping [40]. Analytical variables include the choice of endogenous control, preamplification protocol, and positivity threshold, none of which have been harmonized across institutions. Until an international consensus establishes standardized pre-analytical protocols, universal reference ranges, and scenario-specific cutoff values for each clinical application (diagnosis, monitoring, and surveillance), results from different centers will remain difficult to compare directly.

Whether serum or plasma is preferable remains unresolved. Both have been used successfully in published studies, but direct measurements are not interchangeable, and clinical programs should standardize to one matrix. The practical workflow using the CE-certified kit involves serum collection in separation tubes, coagulation at room temperature for one hour, centrifugation at 2000–3000× *g*, and storage of aliquots at −20 to −80 °C. The four analytical steps, RNA isolation, cDNA synthesis, preamplification, and qRT-PCR, can be completed within a single working day in equipped molecular laboratories [8].

To improve cross-study comparability, we suggest that future reports of circulating miR-371a-3p, as a minimum, specify: the matrix (serum or plasma); the quantification platform (qRT-PCR with preamplification, or ddPCR); the endogenous normalizer used; the preamplification protocol; the derivation and value of the positivity threshold; a hemolysis assessment; and the time from collection to processing.

Practical integration also faces operational hurdles that extend beyond assay chemistry. miR-371a-3p quantification currently requires molecular-laboratory infrastructure and trained personnel that are not universally available in general oncology or urology services, and centralized testing introduces sample-transport and turnaround-time constraints that must be reconciled with real-time clinical decision points, such as pre-cycle chemotherapy assessment or intraoperative frozen-section pathways. Cross-center comparability further depends on external quality-assessment schemes and proficiency testing, which do not yet exist for this analyte, and on consensus positivity thresholds that remain undefined. Until inter-laboratory harmonization, external quality control, and a defined reimbursement pathway are established, adoption is likely to remain confined to specialized centers even where a validated assay is technically available.

Beyond analytical standardization, routine adoption faces economic and equity considerations. Blood-based miR-371a-3p testing has been shown to be cost-competitive with, and potentially cheaper than, surveillance CT [29], but formal cost-effectiveness modeling across clinical pathways is lacking. Access is also uneven: a CE-certified assay is available in Europe, whereas the absence of CLIA-approved testing restricts US access largely to research settings, raising equity concerns for global implementation. Meaningful uptake will ultimately depend on incorporation into major guidelines, which at present remains conditional on prospective marker-guided trial data.

## 11. Prospective Trial Landscape and the Trajectory Toward Guideline Incorporation

Several prospective studies are now maturing and may inform future guideline incorporation. However, most available evidence remains diagnostic/prognostic rather than interventional, and prospective trials testing marker-guided management strategies are still needed. While the Southwest Oncology Group study (S1823/NCI-2019-06177) continues its long-term follow-up of 956 TGCT patients to refine positive predictive values across all clinical stages, other pivotal trials have reached maturity. The Haukeland University Hospital study (NCT04914026) is nearing its late-2026 completion, specifically addressing the marker’s utility in pre-orchiectomy diagnosis and the guidance of retroperitoneal lymph node dissections. Notably, the German Testicular Cancer Study Group trial (DRKS00019223) has concluded, documenting a 100% negative predictive value for early relapse detection in Stage I patients [41]. These findings are complemented by the ANZUP-led CLIMATE 1906 study, which recently reported at ASCO GU 2026 that postoperative miR-371a-3p elevations constitute a high-risk indicator for relapse (HR~10), significantly outperforming traditional risk factors like tumor size and lymphovascular invasion in predicting the need for adjuvant treatment [42].

Current major guidelines have adopted a cautiously supportive stance. The EAU guidelines recognize the marker’s emerging potential and its superior discriminatory capacity compared to established STMs while calling for further validation and laboratory standardization before clinical implementation [22]. The ESMO-EURACAN guidelines similarly highlight the promising data without endorsing routine use. Neither the NCCN guidelines incorporate the marker, reflecting the absence of CLIA-approved testing in the United States. As trial results mature and regulatory pathways are navigated, guideline incorporation is a plausible near-term outcome, although the timeline will depend on the resolution of assay standardization issues and the strength of the prospective data.

## 12. Clinical Utility Summary

**Figure 1:**
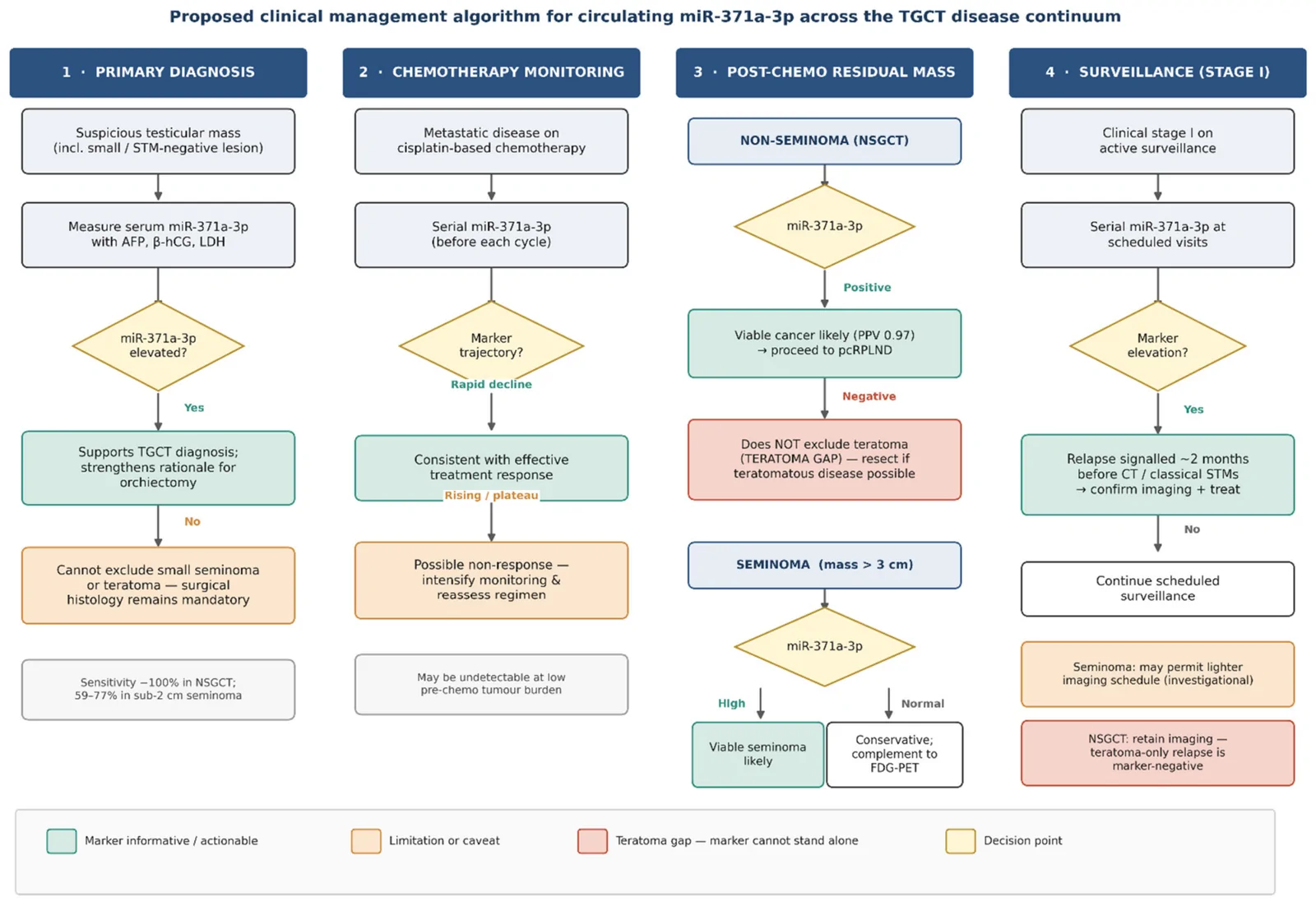
Proposed clinical management algorithm for circulating miR-371a-3p across the TGCT disease continuum. The marker is positioned as an adjunct at four decision points: primary diagnosis of suspicious (including small or STM-negative) masses; response monitoring during cisplatin-based chemotherapy; assessment of post-chemotherapy residual masses in non-seminoma and seminoma; and relapse detection during Stage I surveillance. Red elements denote the teratoma gap, where a negative marker cannot exclude teratomatous disease and imaging or resection remains mandatory. Blue boxes denote disease-specific clinical subgroup headings or management contexts. AFP = alpha-fetoprotein; β-hCG = beta-human chorionic gonadotropin; LDH = lactate dehydrogenase; NSGCT = non-seminomatous germ cell tumor; pcRPLND = post-chemotherapy retroperitoneal lymph node dissection; PPV = positive predictive value; STM = serum tumor marker.

Figure 1 presents the proposed clinical algorithm integrating miR-371a-3p across the disease continuum, and Table 3 maps each clinical application of miR-371a-3p to a concise summary of the current evidence, the principal outstanding research need, and a graded level of supporting evidence. Evidence levels are defined as: Strong (consistent findings across multiple prospective cohorts); Intermediate (prospective or sizeable retrospective data, but limited sample size or a single pivotal study); Preliminary (single small cohort); and Preclinical (in vitro or animal-model data only).

## 13. Conclusions

MiR-371a-3p is among the most promising adjunct circulating biomarkers for testicular germ cell tumors, consistently outperforming AFP, β-hCG, and LDH in diagnostic accuracy across multiple clinical cohorts. Its rapid post-treatment decline and close association with viable tumor burden support potential roles in primary diagnosis, treatment monitoring, relapse detection, and selected residual mass assessment.

However, important limitations remain. MiR-371a-3p does not detect teratoma, limiting its ability to replace imaging or surgery in settings where teratomatous disease is possible. Assay heterogeneity, the lack of universally accepted cutoffs, and incomplete regulatory implementation also restrict routine clinical use. Moreover, most evidence supports diagnostic and prognostic utility rather than validated marker-guided treatment decisions.

Beyond its biomarker role, miR-371a-3p appears biologically relevant in tumor–microenvironment communication, cisplatin resistance, and reproductive biology, although these areas remain largely preclinical or exploratory. Overall, miR-371a-3p is a highly promising adjunct to current TGCT management, but translating it into routine care now depends on a defined set of concrete steps rather than on further confirmatory diagnostic studies. The priorities are, in order: (i) completion and reporting of the maturing prospective marker-guided trials (S1823/NCI-2019-06177, NCT04914026, and the ANZUP-led CLIMATE 1906 study) that test whether marker-driven decisions change outcomes; (ii) international assay harmonization, supported by external quality-assessment schemes and a consensus positivity threshold; (iii) resolution of the discordant data on postoperative marker elevation through studies using harmonized collection timing and thresholds; (iv) regulatory qualification beyond the current CE-marked assay, including a CLIA-approved pathway to address the transatlantic access gap; and (v) validation of a teratoma-specific biomarker to close the single biological limitation that most constrains marker-guided surgical de-escalation. Progress across these five fronts, rather than additional case–control diagnostic series, will determine whether miR-371a-3p enters guideline-endorsed clinical practice.

## Figures and Tables

**Table 2:** Performance of miR-371a-3p for relapse detection in TGCT surveillance.

Author, Year	Total N	N Relapsed	Sensitivity (%)	Specificity (%)	AUC	Matrix/Platform
Dieckmann, 2019 [14]	616	46	82.6	96.1	0.92	Serum/qRT-PCR
Lobo, 2021 [34]	151	34	94.1	NR	NR	Serum/qRT-PCR
Fankhauser, 2022 [35]	33	10	100	100	1.0	Serum/qRT-PCR
Nappi, 2023 [36]	101	35	62.8	100	0.81	Plasma/qRT-PCR

NR = not reported; AUC = area under the receiver operating characteristic curve.

**Table 3:** Precision oncology applications of miR-371a-3p in TGCT: evidence, research priorities, and level of evidence.

Clinical Scenario	Current Evidence	Research Priorities	Evidence Level
Small/ambiguous testicular mass	Sensitivity near 100% in NSGCT; ~59–77% in small seminoma. Specificity 94–100% vs. controls.	Prospective data in STM-negative masses; comparison with outcomes following omission of frozen sections.	Intermediate (retrospective data, small cohorts)
Primary diagnosis	AUC 0.93–0.98 across 12+ studies. Outperforms all classical STMs individually.	International assay standardization; CLIA approval; consensus cutoff values.	Strong (multiple prospective cohorts)
Chemotherapy monitoring	Rapid decline correlates with response; rising levels associated with treatment failure in limited cases.	Prospective adaptive treatment trials; kinetics-based algorithms for regimen modification.	Intermediate (prospective data, small sample sizes for key findings)
Post-CTx residual mass (NSGCT)	Prospective sensitivity 68.9% (burden-dependent: 33% for ≤10% VC, 86% for >50% VC); AUC 0.813; PPV 0.969. Cannot detect teratoma or somatic-type malignancy.	Teratoma-specific marker validation; define criteria for safe surveillance in marker-negative patients; combine with radiomics.	Intermediate (one large prospective study)
Post-CTx residual mass (seminoma)	Elevated levels may predict viable seminoma; preliminary data suggest a potential complement to FDG-PET.	Larger prospective cohorts; optimized seminoma-specific cutoffs.	Preliminary (single small cohort)
Surveillance/relapse detection	Detects relapse median 2 months earlier than CT + classical STMs. Sensitivity 62–100%.	CT frequency reduction trials; define safe marker-triggered imaging intervals.	Intermediate to strong (prospective series, but small sample sizes)
Cisplatin resistance (preclinical)	miR-371a-3p elevation in resistant cell lines; antagomir inhibition restores sensitivity in vitro.	In vivo validation; clinical pharmacology of antagomir delivery; dose-reduction trials.	Preclinical only

CTx = chemotherapy; AUC = area under the receiver operating characteristic curve; NSGCT = non-seminomatous germ cell tumor; FDG-PET = fluorodeoxyglucose positron emission tomography; STM = serum tumor marker.

## Data Availability

No new data were created or analyzed in this study. Data sharing is not applicable to this article.
